# Spirometric traits show quantile-dependent heritability, which may contribute to their gene-environment interactions with smoking and pollution

**DOI:** 10.7717/peerj.9145

**Published:** 2020-05-15

**Authors:** Paul T. Williams

**Affiliations:** Molecular Biophysics & Integrated Bioimaging, Lawrence Berkeley National Laboratory, Berkeley, CA, United States of America

**Keywords:** Pulmonary function, Gene environment interaction, Heritability, Smoking, Pollution, Forced vital capacity, SERPINA1, COPD, Quantile dependent expressivity, Spirometric data

## Abstract

**Background:**

“Quantile-dependent expressivity” refers to a genetic effect that is dependent upon whether the phenotype (e.g., spirometric data) is high or low relative to its population distribution. Forced vital capacity (FVC), forced expiratory volume in 1 second (FEV_1_), and the FEV_1_/FVC ratio are moderately heritable spirometric traits. The aim of the analyses is to test whether their heritability (*h^2^*) is constant over all quantiles of their distribution.

**Methods:**

Quantile regression was applied to the mean age, sex, height and smoking-adjusted spirometric data over multiple visits in 9,993 offspring-parent pairs and 1,930 sibships from the Framingham Heart Study to obtain robust estimates of offspring-parent (β_OP_), offspring-midparent (β_OM_), and full-sib regression slopes (β_FS_). Nonparametric significance levels were obtained from 1,000 bootstrap samples. β_OP_s were used as simple indicators of quantile-specific heritability (i.e., *h*^2^ = 2β_OP_/(1+r_spouse_), where r_spouse_ was the correlation between spouses).

**Results:**

β_OP_ ± standard error (SE) decreased by 0.0009 ± 0.0003 (*P* = 0.003) with every one-percent increment in the population distribution of FEV_1_/FVC, i.e., β_OP_ ± SE were: 0.182 ± 0.031, 0.152 ± 0.015; 0.136 ± 0.011; 0.121 ± 0.013; and 0.099 ± 0.013 at the 10th, 25th, 50th, 75th, and 90th percentiles of the FEV_1_/FVC distribution, respectively. These correspond to *h^2^* ± SEs of 0.350 ± 0.060 at the 10th, 0.292 ± 0.029 at the 25th, 0.262 ± 0.020 at the 50th, 0.234 ± 0.025 at the 75th, and 0.191 ± 0.025 at the 90th percentiles of the FEV_1_/FVC ratio. Maximum mid-expiratory flow (MMEF) *h^2^* ± SEs increased 0.0025 ± 0.0007 (*P* = 0.0004) with every one-percent increment in its distribution, i.e.: 0.467 ± 0.046, 0.467 ± 0.033, 0.554 ± 0.038, 0.615 ± 0.042, and 0.675 ± 0.060 at the 10th, 25th, 50th, 75th, and 90th percentiles of its distribution. This was due to forced expiratory flow at 75% of FVC (FEF75%), whose quantile-specific *h^2^* increased an average of 0.0042 ± 0.0008 for every one-percent increment in its distribution. It is speculated that previously reported gene-environment interactions may be partially attributable to quantile-specific *h^2^*, i.e., greater heritability in individuals with lower FEV_1_/FVC due to smoking or airborne particles exposure vs. nonsmoking, unexposed individuals.

**Conclusion:**

Heritabilities of FEV_1_/FVC, MMEF, and FEF75% from quantile-regression of offspring-parent and sibling spirometric data suggest their quantile-dependent expressivity.

## Introduction

Forced vital capacity (FVC), forced expiratory volume in 1 s (FEV_1_), and the FEV_1_/FVC ratio are the most commonly measured spirometric traits (Global initiative for Chronic Obstructive Lung Disease, 2020; Wood, Tan & Stockley, 2009). FVC approximates lung volume and is a strong predictor of all-cause mortality (Global initiative for Chronic Obstructive Lung Disease, 2020; Wood, Tan & Stockley, 2009). Reduced FVC in the absence of reduced FEV_1_/FVC indicates a tendency towards a restrictive ventilatory defect. Low FEV_1_/FVC (e.g.,  < lower limit of normal or z-score < -1.64) identifies patients with airflow obstruction, and low FEV_1_ (as a percentage of predicted values or as z-scores < -1.64) their obstruction severity. The Global Initiative for Chronic Obstructive Lung Disease (GOLD) defines chronic obstructive pulmonary disease (COPD) as a post-bronchodilator FEV_1_/FVC ratio < 0.70 and FEV_1_ ≥80% of predicted (GOLD I), 50% ≤ FEV_1_ <80% (GOLD II), 30% ≤ FEV_1_ <50% (GOLD III), and FEV_1_ <30% predicted (GOLD IV) (Global initiative for Chronic Obstructive Lung Disease, 2020). Maximum mid-expiratory flow (MMEF) and forced expiratory flows at X% of FVC (i.e., FEF25%, FEF50% and FEF75%) may assess small airway caliber especially in case of a normal FVC (Wood, Tan & Stockley, 2009), although its use in diagnosing small airway disease in individual patients is discouraged (Quanjer et al., 2014). Peak expiratory flow (PEF) is the maximum speed of expiration.

Heritability estimates range widely across family and twins studies, from 0.09 to 0.68 for FEV_1_ (mean 0.39), 0.20 to 0.78 for FVC (mean 0.45), and 0.16 to 0.64 for the FEV_1_/FVC ratio (mean 0.36) (Devor & Crawford, 1984; Coultas et al., 1991; Klimentidis et al., 2013; Redline et al., 1989; Wilk et al., 2000; Tarnoki et al., 2013; Hukkinen et al., 2011; Hallberg et al., 2010; DeMeo et al., 2004; McClearn et al., 1994; Palmer et al., 2001; Astemborski, Beaty & Cohen, 1985; Beaty et al., 1987; Lewitter et al., 1984; Cotch, Beaty & Cohen, 1990; Chen et al., 1996; Chen et al., 1997; Givelber et al., 1998; Ingebrigtsen et al., 2011; Joost et al., 2002; Tian et al., 2017; Yamada et al., 2015). Prior segregation analyses of pulmonary function generally favor its polygenic inheritance (Chen et al., 1996; Chen et al., 1997; Givelber et al., 1998). Forty-nine genetic loci have been significantly related to pulmonary function in meta-analyses of 38,199 individuals of European ancestry from 17 genome-wide association studies (GWAS) (Loth et al., 2014; Soler Artigas et al., 2015; Hancock et al., 2010; Repapi et al., 2010; Soler Artigas et al., 2011). As in other complex traits (Manolio et al., 2009), only a small proportion of the heritability is attributable to known single nucleotide polymorphisms (SNPs): 4.0% of the additive polygenic variance for FEV_1_, 5.4% for FEV_1_/FVC, and 3.2% for FVC (Soler Artigas et al., 2015). Estimates of heritability based on genome-wide SNPs are more consistent with pedigree-based estimates (Klimentidis et al., 2013; Yamada et al., 2015).

Smoking is the strongest environmental cause for reduced pulmonary function. The average decline of lung function with age is approximately 50% greater in smokers than nonsmokers (Gottlieb, 1999). Gene-environment interactions with smoking and airborne particle exposure have been found in multiple reports (Curjuric et al., 2012; Hallberg et al., 2010; He et al., 2004; Zhai et al., 2007; Mehta et al., 2014; Sigsgaard et al., 2000; Kim et al., 2015) and used to improve power for identifying genetic variants associated with pulmonary function (Hancock et al., 2012). Having a first-degree relative with COPD is associated with a 13% mean reduction in FEV_1_ in smokers but not in nonsmokers (Aschard et al., 2017). Moreover, COPD risk is two to four times greater in smokers having a first-degree relative with COPD than in smokers that do not (Walter, Gottlieb & O’Connor, 2000). Among persons with severe a-1 antitrypsin (AAT) deficiency, mortality occurs earlier and pulmonary function declines more rapidly with age in smokers than nonsmokers (Walter, Gottlieb & O’Connor, 2000). Reductions in FEV_1_/FVC with smoking are reported to be greater among individuals who are genetically predisposed to lower FEV _1_/FVC (Aschard et al., 2017). Gene-environment interactions have also been reported between smoking and chronic bronchitis (Hallberg et al., 2008), a condition indicating reduced FEV_1_ (Vestbo, Prescott & Lange, 1996).

Elsewhere it has been shown that quantile-specific effects play a fundamental role in the genetics of body weight, lipoprotein concentrations, and coffee intake (quantile-dependent penetrance or expressivity) while not affecting other traits such as height (Williams, 2012; Williams, 2020c; Williams, 2020b; Williams, 2020a). It is not known whether quantile-specific genetic effects apply to pulmonary function. The aforementioned twin and family studies (Devor & Crawford, 1984; Coultas et al., 1991; Klimentidis et al., 2013; Redline et al., 1989; Wilk et al., 2000; Tarnoki et al., 2013; Hukkinen et al., 2011; Hallberg et al., 2010; DeMeo et al., 2004; McClearn et al., 1994; Palmer et al., 2001; Astemborski, Beaty & Cohen, 1985; Beaty et al., 1987; Lewitter et al., 1984; Cotch, Beaty & Cohen, 1990; Chen et al., 1996; Chen et al., 1997; Givelber et al., 1998; Ingebrigtsen et al., 2011; Joost et al., 2002; Tian et al., 2017; Yamada et al., 2015), segregation analyses (Chen et al., 1996; Chen et al., 1997; Givelber et al., 1998), and GWAS (Loth et al., 2014; Soler Artigas et al., 2015; Hancock et al., 2010; Repapi et al., 2010; Soler Artigas et al., 2011) are all based on the assumption that the genetic and other inherited effects are the same throughout the distributions of pulmonary function, i.e., the same whether pulmonary function is high or low relative to the population distribution. Quantile regression was therefore applied to spirometric data from the Framingham Study (Dawber, Meadors & Moore, 1951; Kannel et al., 1979; Splansky et al., 2007) to assess whether quantile-dependent expressivity affects its heritability.

## Population and Methods

The Framingham Study data were obtained from the National Institutes of Health FRAMCOHORT, GEN3, FRAMOFFSPRING Research Materials obtained from the NHLBI Biologic Specimen and Data Repository Information Coordinating Center. Approval for the analyses of these data were obtained from the Committee for the Protection of Human Subjects at Lawrence Berkeley National Laboratory, Department of Energy, Berkeley, California (Protocol Title: Gene-environment interaction vs. quantile-dependent penetrance of established SNPs. APPROVAL NUMBER: 107H021-13MR20).

The Original (generation 1) Framingham Cohort consisted of 5,209 men and women between the ages of 30 and 62 from the town of Framingham, Massachusetts who were recruited and examined between 1948 and 1953 and re-examined biannually thereafter (Dawber, Meadors & Moore, 1951). The Offspring Cohort (generation 2) consisted of 5,124 adult offspring of the original participants and their spouses who were first examined between 1971 and 1975, reexamined eight years later and then every three to four years thereafter (Kannel et al., 1979). Children of the Offspring Cohort were recruited to form the Third Generation Cohort, which was examined twice (Splansky et al., 2007). Participants used in the current analyses were at least 16 years of age and self-identified as white or non-Hispanic in the Offspring and Third Generation Cohorts (race and ethnicity were not requested in the Original Cohort, but reported to be overwhelmingly white). FVC and FEV_1_ were measured at exams 5, 6, 16, 17 and 19 of the Original Cohort; exams 3 and 5-8 of the Offspring Cohort; and exams 1 and 2 of the Third Generation Cohort. MMEF, FEF25%, FEF50% and FEF75% were measured at exams 16, 17 and 19 of the Original; exams 3, 5-8 of the Offspring; and exams 1 and 2 of the Third Generation Cohort. PEF was measured at exam 19 of the Original Cohort, exams 3 and 5-8 of the Offspring Cohort, and exams 1 and 2 of Third Generation Cohort. There were no exclusions for chronic bronchitis, pulmonary disease, COPD, emphysema, or asthma.

Spirometry methodology and quality changed over the history of the Framingham Heart Study, particularly prior to 1980 (Joost et al., 2002; Rice et al., 2013; Liao, Lin & Christiani, 2013). Spirometry at examinations 5 (conducted between 1958-1959) and 6 (1960 and 1961) of the Original Cohort used a 13.5L Collins water-sealed bell spirometer. Each participant performed three FVC maneuvers while standing. FEV_1_ and FVC were measured by hand from the chymograph tracings using the method of back extrapolation. Only the maneuver with the greatest FVC was recorded in the chart. Although these maneuvers were performed before the introduction of the American Thoracic Society guidelines for standardization of spirometry, a review of spirograms showed that 80% of the tracings met acceptability guidelines for smoothness of curve and forceful initial push. Raw values were multiplied by 1.10 to correct for body temperature, ambient pressure, saturated with water (BTPS), assuming a clinic temperature of 20°C.

Spirometry for later exams of the Original Cohort and examination 3 (1984–1987) of the Offspring Cohort used the 6L Collins water-sealed bell spirometer connected to a microprocessor that provided automatic correction for BTPS. At Offspring Cohort examination 5 (Cycle 5, 1992–1995), the spirometer was interfaced with lung function software that provided real-time quality assurance. Exams 6 and 7 of the Offspring Cohort used a Collins Survey II spirometer with pulmonary function data acquisition and quality control software (S&M Instruments, Doylestown, PA) that was calibrated daily. Spirometric data for the Third Generation Cohort were obtained on a Collins CPL system (Spire Health, Inc., Longmont, CO) that was calibrated daily. FVC maneuvers were performed standing while wearing nose clips and repeated until at least three acceptable spirograms were obtained, up to a maximum of eight spirograms, using American Thoracic Society standards to identify acceptable spirograms. The largest FEV_1_ and the largest FVC from among all acceptable maneuvers, and the FEV_1_/FVC ratio from the maneuver with the largest sum of FEV_1_ + FVC, were used in this analysis.

### Applied definitions

Current cigarette smokers were defined as responding yes to the question “If ever smoked cigarettes regularly, have you smoked cigarettes regularly in the past year?” or reporting a positive number in response to the question “If you ever smoked cigarettes regularly, how many cigarettes do you smoke per day now?” Pulmonary function measures were not significantly affected by reported pipe or cigar smoking in these data and were therefore included in the never smoked cigarette group (Givelber et al., 1998). Cigarette smokers who reported not inhaling had about half the effect on lung function than those that inhaled, and were included in the cigarette smokers. Former smoker were defined as noncurrent smokers who were classified as a current smoker at any prior visit or who reported a history of having smoked.

### Statistical analyses

Adjustment was performed separately in the Original, Offspring and Third Generation Cohorts which should partially correct for differences in methodology over time and differences between cohorts (e.g., Offspring Cohort being taller and heavier than members of the Original Cohort). All observations were adjusted for sex, age (age and age^2^ separately in men and women), height (separately in men and women), and whether the participant currently smoked cigarettes or previously smoked cigarettes. Individual participant values were taken as the average of the residuals over all available exams (i.e., the average of up to five exams for the Original Cohort, up to five exams for the Offspring Cohort, and up to two exams for the Third Generation Cohort). Population-based studies suggest that FEV_1_ and FVC are approximately normally distributed in the general population (Palmer et al., 2001; Dockery et al., 1985).

Offspring-parent correlations and regression slopes were computed using offspring and parents from: (1) Offspring and Original Cohorts, and (2) Third Generation and Offspring Cohorts. Offspring-parent correlations and regression slopes were computed by assigning a weight of one-half to the father-child and one-half to the mother-child pair (if both parents available), or assigning a weight of one to the parent–child pair if only one parent was available. Sibling correlations and full-sib regression slopes were obtained by forming all k_*i*_(k_*i*_-1) sibpair combinations for the k_*i*_ siblings in sibship i and assigning equal weight to each individual (Karlin, Cameron & Williams, 1981). The number of degrees of freedom for the standard error was Σk_*i*_-2 for offspring-parent regression slopes and correlations, and Σ(k_*i*_-1) for sibship correlations and regression slopes, where the summation is taken over all *i*, *i* = 1,…, N families. Heritability in the narrow sense (*h*^2^**) was estimated as *h*^2^ = 2*β*_*OP*_/(1+r_spouse_) from the offspring-parent regression slope (*β*_*OP*_), as *h*^2^ = *β*_*OM*_ from the offspring-midparental regression slope (*β*_*OM*_), and as *h*^2^ = [(1 + 8*r*_spouse_β_*FS*_)^0.5^ − 1]∕(2*r*_spouse_) from full-sibs regression slopes (β_*FS*_), where r_spouse_ is the spouse correlation (Falconer & Mackay, 1996). Means are presented with their associated standard deviations (SD), while slopes and *h*^2^** are presented ± standard error (±SE). “Quantile-specific heritability” refers to the heritability statistic (*h*^2^**), whereas “quantile-dependent expressivity” is the biological phenomenon of the trait expression being quantile-dependent.

**Figure 1:**
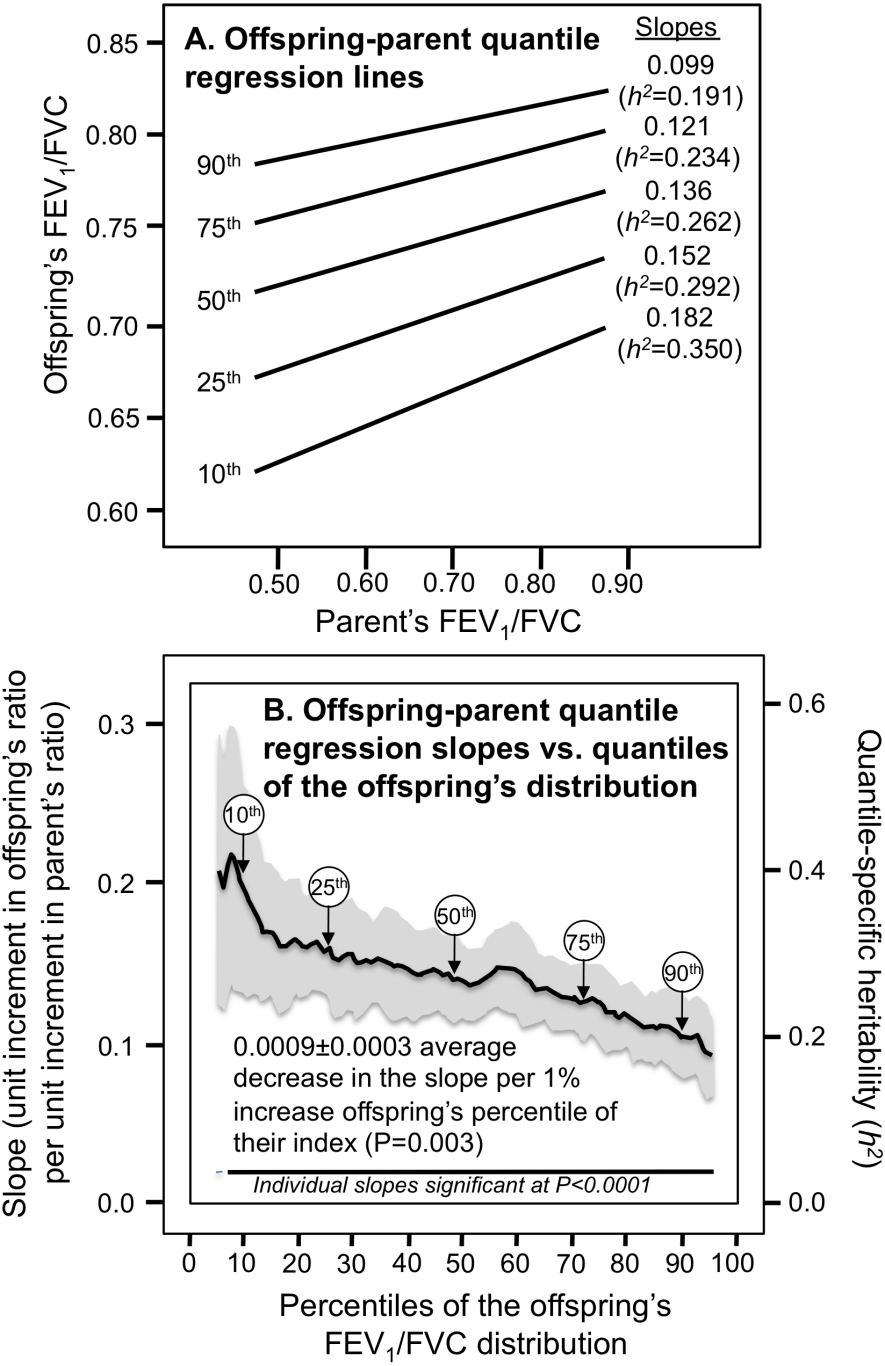
Offspring-parent regression slopes (*β*_*OP*_) for selected quantiles of the offsprings’ FEV1/FVC ratio (10th, 25th, 50th, 75th, 90th). (A) Offspring-parent regression slopes (*β*_*OP*_) for selected quantiles of the offspring’s FEV_1_/FVC distribution (10th, 25th, 50th, 75th, 90th) for quantile regression analyses of 6,223 offspring, showing increasing regression slope with decreasing percentiles of the offspring’s distribution. (B) The slopes from A were included with those of other quantiles to create the quantile-specific heritability plot. The shaded area presents the 95% confidence intervals for the individual slopes at each quantile. Quantile-specific heritability (*h*^2^) was calculated as 2*β*_*OP*_∕(1 + *r*_spouse_) where *r*_spouse_ = 0.04.

Simultaneous quantile regression is a well-developed statistical method (Koenker & Hallock, 2001; Gould, 1992) that estimates the regression coefficients for multiple quantiles using linear programming to minimize the sum of asymmetrically weighted absolute residuals, and bootstrap resampling to estimate their corresponding variances and covariances. Simultaneous quantile regression was performed using the sqreg command of Stata (version. 11, StataCorp, College Station, TX) with one thousand bootstrap samples drawn to estimate the variance–covariance matrix for the 91 quantile regression coefficients between the 5th and 95th percentiles, and the post-estimation procedures (test and lincom) to test linear combinations of the slopes after estimation with Σk_*i*_-2 degrees of freedom for offspring-parent regression slopes (*β*_*OP*_) and Σ(k_*i*_-1) degrees of freedom for full-sib regression slopes (*β*_*FS*_). Quantile-dependent expressivity was assessed by: (1) estimating quantile-specific β-coefficient for the 5th, 6th,…, 95th percentiles of the sample distribution using simultaneous quantile regression (Fig. 1, the <5th and >95th percentiles ignored because they were thought to be less stable); (2) plotting the quantile-specific β coefficients vs. the quantile of the trait distribution; and (3) testing whether the resulting graph is constant, or changes as a linear, quadratic, or cubic function of the percentile of the trait distribution using orthogonal polynomials (Winer, Brown & Michels, 1991).

Data availability:** The data that support the findings of this study are available from dbGaP (dbGaP genotypes & phenotypes, 2020a). Restrictions apply to the availability of these data, which were used under license for this study. Data are available with the permission of dbGaP with appropriate human use approval. The public summary-level phenotype data may be browsed at the dbGaP study home page (dbGaP genotypes & phenotypes, 2020b).

## Results

Table 1 presents the characteristics of the sample, Table 2 presents standard and quantile-regression analyses of spouses, offspring and parents, and among siblings.

**Table 1:** Sample characteristics.

	Parents	Offspring
Sample	4601	6231
Male,%	47.3	47.0
Height, m	1.66 (0.09)	1.69 (0.09)
Ever smoked, %	68.4	51.4
Age, years	59.4 (8.2)	48.2 (11.4)
FVC, l	3.34 (0.95)	4.24 (1.06)
FEV_1_, l	2.46 (0.73)	3.22 (0.86)
FEV_1_/FVC	0.74 (0.09)	0.76 (0.08)
FEF25%, l/s	5.38 (2.09)	6.83 (2.03)
FEF50%, 1/s	2.91 (1.31)	3.88 (1.41)
FEF75%, l/s	0.74 (0.44)	1.21 (0.61)
MMEF, l/s	1.98 (0.92)	2.91 (1.12)
PEF, l/s	7.11 (2.32)	8.44 (2.17)

**Notes.**

Means (standard deviations) are presented. Sample size is for FVC and varies between variables (see figure legends).

**Table 2:** Traditional (least squares) and quantile regression analyses of spirometric traits between related individuals.^a^

	Least-squares regression analysis			Quantile regression analysis			
	Correlation	Least-squares regression slope		Increase in slope per 1% increase in the offspring or sibling distribution		Difference in slope between the 90th and 10th percentiles	
		Slope ± SE	*P*^b^	Slope ± SE	*P*^c^	Difference ± SE	*P*^d^
FEV_1_/FVC							
Spouse	0.04	0.047 ± 0.018	0.007	−0.0005 ± 0.0005	0.37	−0.008 ± 0.077	0.91
Offspring-parent	0.17	0.134 ± 0.010	<10^−15^	−0.0009 ± 0.0003	0.003	−0.083 ± 0.032	0.01
Offspring-midparent	0.24	0.269 ± 0.018	<10^−15^	−0.0021 ± 0.0005	4.1 × 10^−5^	−0.192 ± 0.059	0.001
Full-sibling	0.27	0.266 ± 0.017	<10^−15^	−0.0022 ± 0.0005	2.1 × 10^−6^	−0.200 ± 0.040	5.6 × 10^−7^
FVC							
Spouse	0.08	0.109 ± 0.033	0.001	0.0008 ± 0.0009	0.41	0.049 ± 0.089	0.58
Offspring-parent	0.26	0.263 ± 0.013	<10^−15^	−0.0002 ± 0.0003	0.43	−0.031 ± 0.027	0.24
Offspring-midparent	0.32	0.463 ± 0.022	<10^−15^	−0.0007 ± 0.0006	0.23	−0.050 ± 0.059	0.4
Full-sibling	0.32	0.321 ± 0.017	<10^−15^	0.0001 ± 0.0004	0.76	−0.001 ± 0.039	0.99
FEV_1_							
Spouse	0.08	0.118 ± 0.034	0.0005	−0.0010 ± 0.0008	0.23	−0.073 ± 0.090	0.40
Offspring-parent	0.23	0.227 ± 0.012	<10^−15^	−0.0003 ± 0.0003	0.29	−0.026 ± 0.034	0.44
Offspring-midparent	0.30	0.407 ± 0.022	<10^−15^	−0.0007 ± 0.0006	0.22	−0.009 ± 0.058	0.88
Full-sibling	0.28	0.282 ± 0.017	<10^−15^	−0.0006 ± 0.0004	0.17	−0.070 ± 0.045	0.12
PEF							
Spouse	0.05	0.078 ± 0.045	0.08	0.0001 ± 0.0012	0.92	−0.047 ± 0.111	0.67
Offspring-parent	0.19	0.180 ± 0.014	<10^−15^	0.0003 ± 0.0003	0.32	0.017 ± 0.034	0.61
Offspring-midparent	0.25	0.336 ± 0.026	<10^−15^	0.0006 ± 0.0006	0.34	0.052 ± 0.055	0.34
Full-sibling	0.23	0.228 ± 0.017	<10^−15^	0.0003 ± 0.0004	0.46	0.063 ± 0.046	0.17
MMEF							
Spouse	0.04	0.049 ± 0.038	0.20	−0.0012 ± 0.0009	0.20	−0.106 ± 0.097	0.28
Offspring-parent	0.25	0.285 ± 0.015	<10^−15^	0.0013 ± 0.0004	0.0004	0.108 ± 0.037	0.003
Offspring-midparent	0.35	0.550 ± 0.028	<10^−15^	0.0014 ± 0.0008	0.06	0.137 ± 0.071	0.05
Full-sibling	0.30	0.296 ± 0.017	<10^−15^	0.0009 ± 0.0005	0.04	0.049 ± 0.049	0.32
FEF25%							
Spouse	0.07	0.101 ± 0.043	0.02	−0.0007 ± 0.0011	0.52	−0.097 ± 0.105	0.36
Offspring-parent	0.21	0.200 ± 0.013	<10^−15^	−0.0002 ± 0.0003	0.45	−0.024 ± 0.032	0.47
Offspring-midparent	0.28	0.367 ± 0.024	<10^−15^	−0.0005 ± 0.0006	0.35	−0.092 ± 0.060	0.13
Full-sibling	0.24	0.241 ± 0.017	<10^−15^	−0.0007 ± 0.0004	0.12	−0.032 ± 0.035	0.36
FEF50%							
Spouse	0.06	0.077 ± 0.037	0.04	−0.0007 ± 0.0009	0.41	−0.124 ± 0.080	0.12
Offspring-parent	0.25	0.253 ± 0.013	<10^−15^	0.0011 ± 0.0003	0.002	0.079 ± 0.037	0.03
Offspring-midparent	0.37	0.514 ± 0.024	<10^−15^	0.0010 ± 0.0006	0.10	0.060 ± 0.060	0.32
Full-sibling	0.28	0.284 ± 0.017	<10^−15^	0.0008 ± 0.0004	0.05	0.055 ± 0.044	0.21
FEF75%							
Spouse	0.01	0.015 ± 0.038	0.70	−0.0012 ± 0.0011	0.27	−0.114 ± 0.097	0.24
Offspring-parent	0.20	0.249 ± 0.017	<10^−15^	0.0021 ± 0.0004	1.8 × 10^−6^	0.153 ± 0.048	0.002
Offspring-midparent	0.28	0.507 ± 0.033	<10^−15^	0.0035 ± 0.0009	0.0001	0.228 ± 0.090	0.01
Full-sibling	0.27	0.269 ± 0.017	<10^−15^	0.0019 ± 0.0005	0.0002	0.147 ± 0.052	0.005

**Notes.**

a See figure legends for sample sizes.

b Significance determined from standard least-squares regression giving each offspring equal weight.

c Significance determined from linear contrast from the variance–covariance matrix determined from 1000 bootstrap samples.

d Significance determined from difference in coefficients between the 10th and 90th quantiles and their associated standard errors (±SE) and covariance determined from 1,000 bootstrap samples. Significance levels are those reported by Stata, but it is important not to interpret significance levels much less than *P* < 0.001 literally but rather as indicators of strong significance.

### Classical estimates of heritability

When adjusted for assortative mating, and ignoring shared environment and dominance in sibs, the classical estimates of heritability (*h*^2^**) from offspring-parent regression slopes and full-sib correlations respectively, were: (1) 0.49 and 0.61 for FVC; (2) 0.42 and 0.54 for FEV_1_; (3) 0.26 and 0.53 for the FEV_1_/FVC ratio; (4) 0.34 and 0.45 for PEF; (5) 0.55 and 0.59 for MMEF; and (6) 0.37 and 0.46 for FEF25%, 0.48 and 0.54 for FEF50%, and 0.49 and 0.54 for FEF75%.

### FEV_1_/FVC ratio

Figure 1A presents the offspring-parent regression slopes (β_*OP*_) for selected quantiles of the offspring’s FEV_1_/FVC ratio. The slopes became progressively weaker (i.e., less steep) with increasing quantiles of the index (*P* = 0.003). These quantile-specific regression slopes were included with those of other quantiles to create the quantile-specific heritability function in Fig. 1B, i.e., where the offspring-parent slopes (*Y*-axis) are plotted as a function of the quantile of the offspring’s sample distribution (*X*-axis). Specifically, the *Y*-axis represents the slope at the 5th quantile of the index, the 6th quantile of the index,…, and the 95th quantiles of the offspring’s index. The shaded area presents the 95% confidence intervals for the individual slopes at each quantile. The figure shows that each unit increment in the parents’ value was associated with an offsprings’ increase of (slope ± SE) 0.182 ± 0.031 units at the 10th percentile of the offspring’s distribution (*P* = 6.4 × 10^−9^), 0.152 ± 0.015 increase at the 25th percentile (*P* < 10^−15^), 0.136 ± 0.011 increase at the 50th percentile (*P* < 10^−15^), 0.121 ±0.013 increments at the 75th percentile (*P* < 10^−15^), and 0.099 ± 0.013 increment at the 90th percentile (*P* < 10^−15^). These correspond to *h*^2^** ± SEs of 0.350 ±0.060 at the 10th, 0.292 ± 0.029 at the 25th, 0.262 ± 0.020 at the 50th, 0.234 ±0.026 at the 75th, and 0.191 ± 0.025 at the 90th percentiles of the FEV_1_/FVC ratio. If the offspring-parent slope for the FEV_1_/FVC ratio was the same for all offspring quantiles as traditionally assumed, then Fig. 1A would display parallel regression lines, and Fig. 1B would present a simple horizontal line. In fact, the graph shows that the slopes became progressively weaker with increasing quantiles of its offspring’s distribution, such that on average each 1-percent increase in the offspring’s distribution was associated with a 0.0018 unit linear decrease in *h*^2^** (*P* = 0.003).

**Figure 2:**
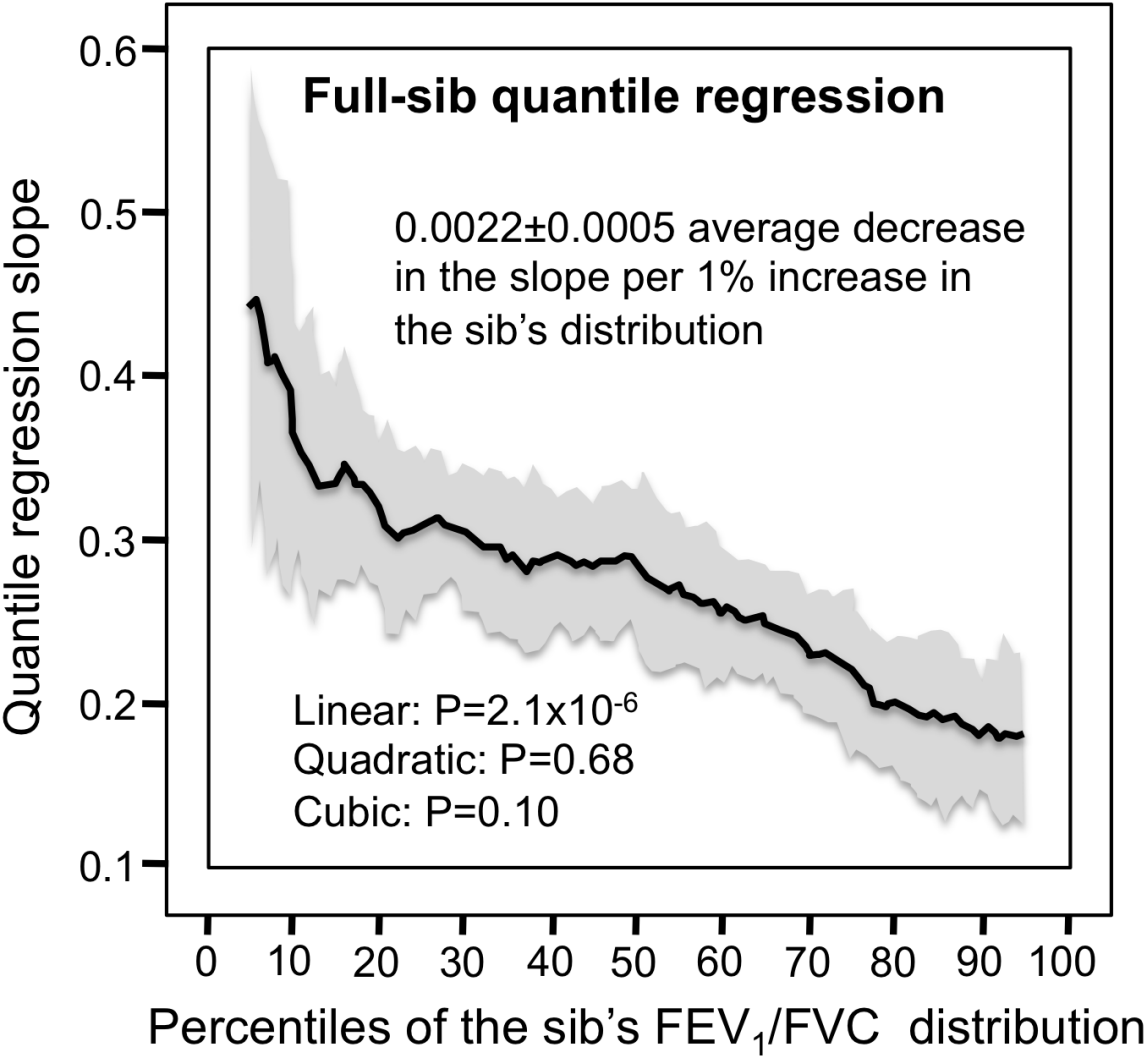
Full-sib regression slopes for FEV1/FVC ratio. Full-sib regression slopes (*β*_*FS*_) for 5,122 sibling from 1,930 sibships showing higher full-sib regression slope for the lower percentiles of the siblings’ FEV _1_/FVC distribution. The shaded area presents the 95% confidence intervals for the individual slopes at each quantile from 1,000 bootstrapped samples. The significance levels of the linear, quadratic and cubic component of the quantile-specific slope function were computed from orthogonal contrasts.

Heritability was nearly 50% lower at the 90th than at the 10th percentile of offspring’s ratio. The *h*^2^** difference between the most healthy and least healthy index (i.e., 90th −10th percentile): was −0.159 ± 0.062 (*P* = 0.01). Figure 2 shows that the quantile-specific heritability for the FEV_1_/FVC ratio was also evident from the regression slopes between sibling. Specifically, the full-sib regression slope: (1) declined 0.0022 ± 0.0005 (*P* = 2.1 × 10^−6^) for each percentile increase in the distribution of the ratio, and (2) was 50% lower at the 90th (0.181 ± 0.021) than the 10th percentile (0.380 ± 0.037) of the sibs’ distribution. The offspring-parent and full-sib slopes were statistically significant (*P* < 10^−6^) at every percentile between the 5th and the 95th percentiles of the offspring’s distribution.

### FVC and FEV1

**Figure 3:**
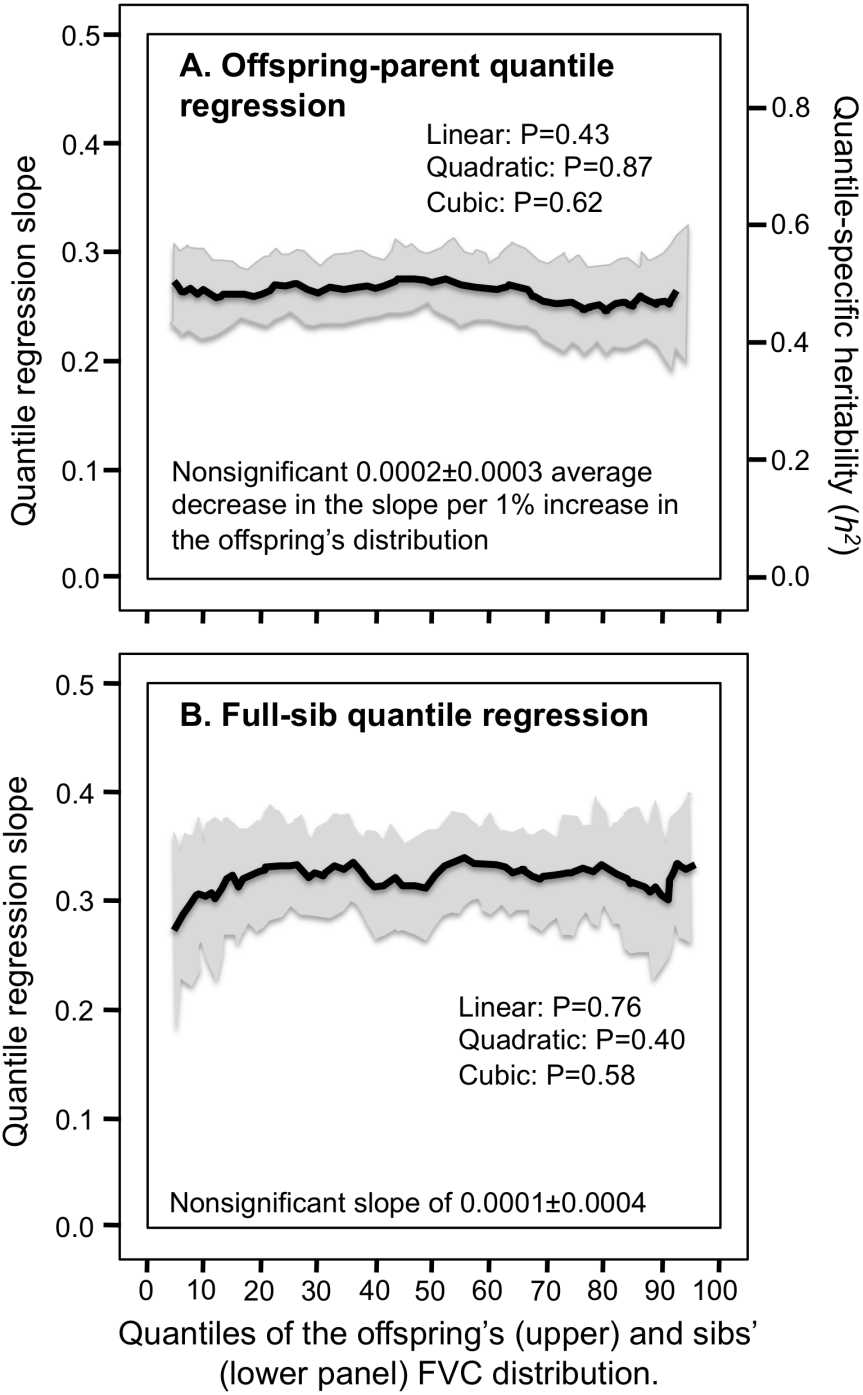
Offspring-parent and full-sib quantile regression slopes for forced vital capacity (FVC). (A) Offspring-parent (*N* = 6,231 offspring) and (B) full-sib quantile regression slopes (5,122 offspring in 1,930 sibships) for FVC. The shaded area presents the 95% confidence intervals for the slopes at each quantile from 1,000 bootstrapped samples. The nonsignificant linear, quadratic and cubic component of the quantile-specific heritability (A) and slope functions (B) were computed from orthogonal contrasts. Quantile-specific heritability (*h*^2^) was calculated as 2*β*_*OP*_∕(1 + *r*_spouse_) where *r*_spouse_ = 0.08.

**Figure 4:**
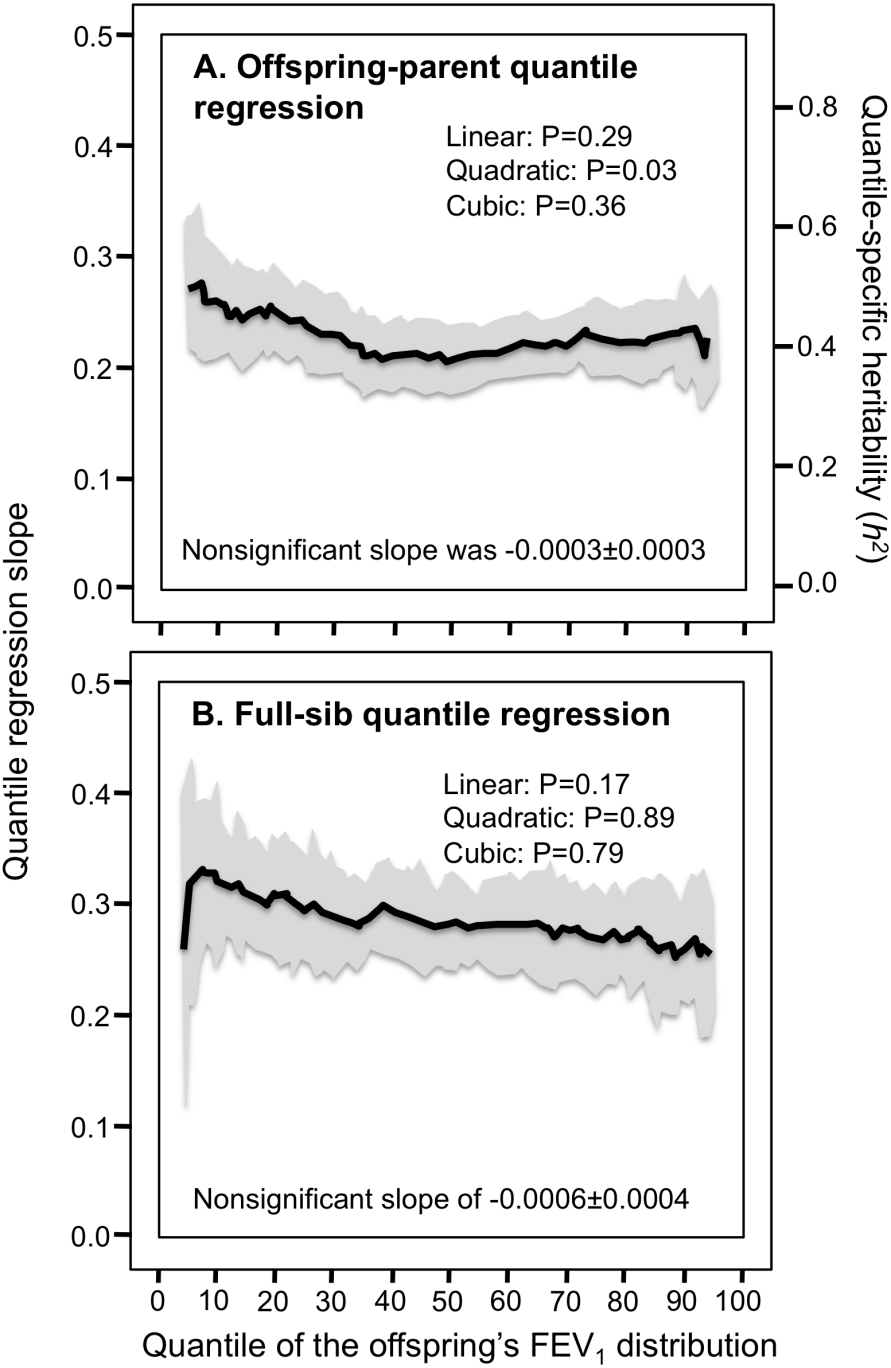
Offspring-parent and full-sib quantile regression for forced expiratory volume at 1 second (FEV _1_). (A) Offspring-parent (*N* = 6,223 offspring) and (B) full-sib quantile regression slopes (*N* = 5,122 offspring in 1,930 sibships) for FEV _1_. The shaded area presents the 95% confidence intervals for the slopes at each quantile from 1,000 bootstrapped samples. Significance for linear, quadratic and cubic component of the quantile-specific heritability and slope functions were computed from orthogonal contrasts. Quantile-specific heritability (*h*^2^) was calculated as 2*β*_*OP*_∕(1 + *r*_spouse_) where *r*_spouse_ = 0.08.

**Figure 5:**
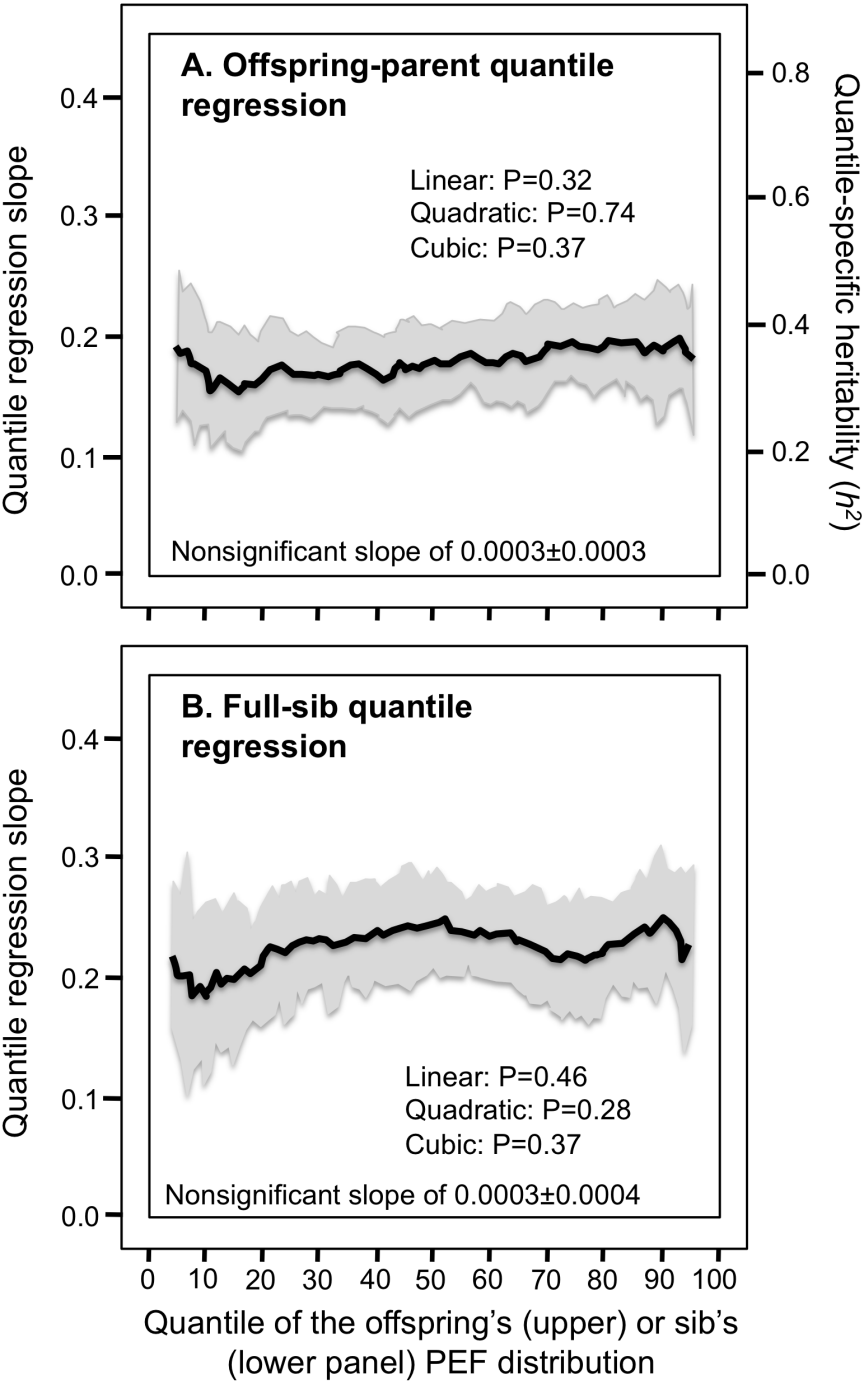
Offspring-parent and full-sib quantile regression slopes for peak expiratory flow (PEF). (A) Offspring-parent (*N* = 4,686 offspring) and (B) full-sib quantile regression slopes (*N* = 5,122 offspring in 1,930 sibships) for PEF. The shaded area presents the 95% confidence intervals for the slopes at each quantile from 1,000 bootstrapped samples. The nonsignificant linear, quadratic and cubic component of the quantile-specific heritability and slope function were computed from orthogonal contrasts. Quantile-specific heritability (*h*^2^) was calculated as 2*β*_*OP*_∕(1 + *r*_spouse_) where *r*_spouse_ = 0.05.

Consistent with the classical model, Table 2 and Figs. 3A and 3B show that the parent–offspring and full-sib regression slopes for FVC were significant and remained relatively constant for all quantiles between the 5th and 95th percentile of their distribution. Figures 4A and 4B and Figs. 5A and 5B show that the slopes for FEV_1_ and PEF were also generally constant, albeit FEV_1_ exhibited some convexity.

### MMEF and FEF

**Figure 6:**
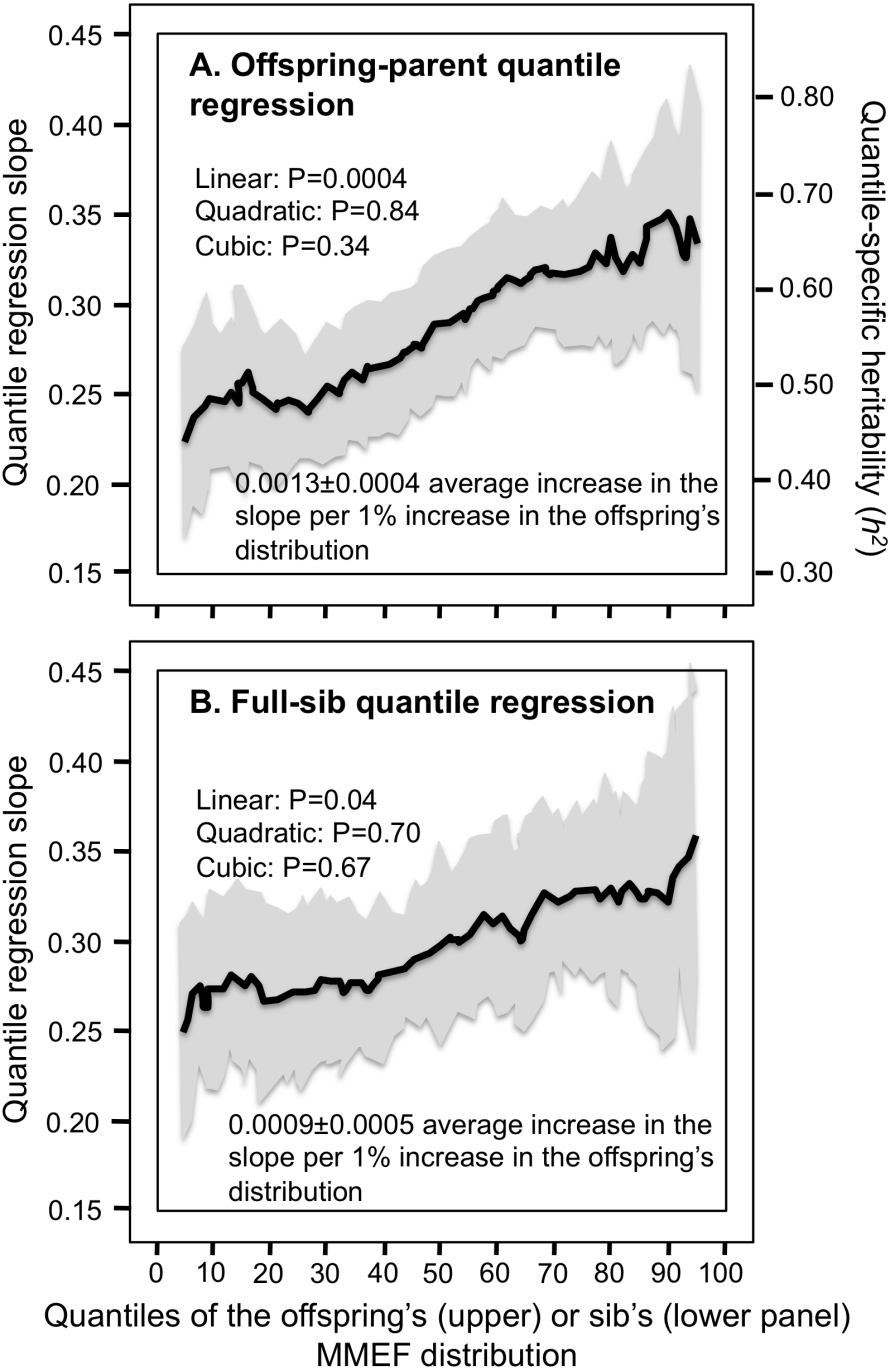
Offspring-parent and full-sib quantile regression for maximum-mid expiratory flow (MMEF). (A) Offspring-parent (*N* = 5,497 offspring) and (B) full-sib quantile regression slopes (*N* = 5,122 offspring in 1,930 sibships) for MMEF. The shaded area presents the 95% confidence intervals for the slopes at each quantile from 1,000 bootstrapped samples. The nonsignificant linear, quadratic and cubic component of the quantile-specific heritability function were computed from orthogonal contrasts. Quantile-specific heritability (*h*^2^) was calculated as 2*β*_*OP*_∕(1 + *r*_spouse_) where *r*_spouse_ = 0.04.

**Figure 7:**
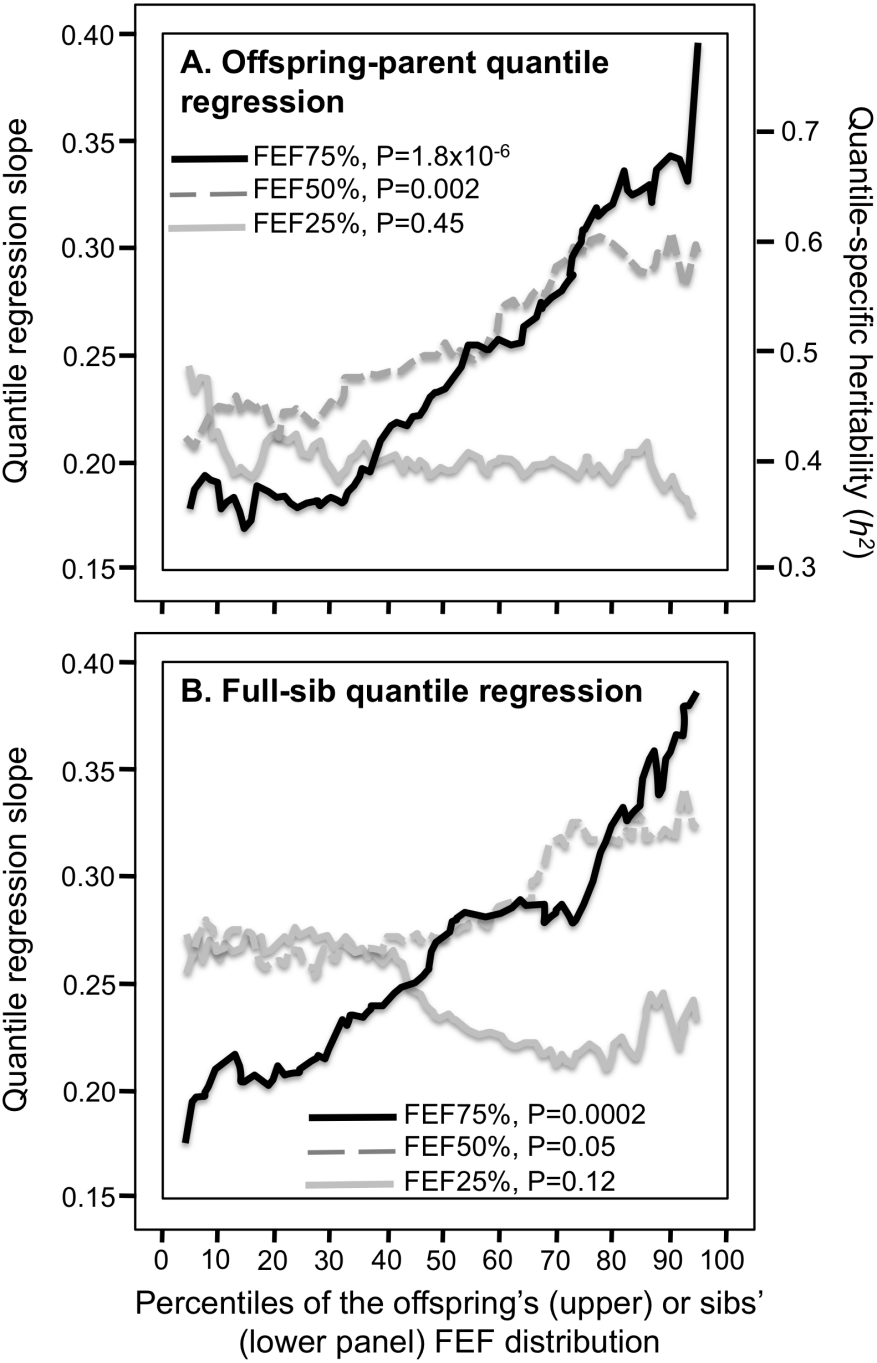
Offspring-parent and full-sib quantile regression slopes for FEF25%, FEF50% and FEF75%. (A) Offspring-parent (*N* = 5,497 offspring) and (B) full-sib quantile regression slopes (5,122 offspring in 1,930 sibships) for FEF25%, FEF50% and FEF75%. The shaded area presents the 95% confidence intervals for the slopes at each quantile from 1,000 bootstrapped samples. The nonsignificant linear, quadratic and cubic component of the quantile-specific heritability function were computed from orthogonal contrasts. Quantile-specific heritability is for forces expired flow at 75% (*r*_spouse_ = 0.01), those for 50% (*r*_spouse_ = 0.06) and 25% (*r*_spouse_ = 0.07) would be slightly lower.

Offspring-parent and full-sib regression slopes for MMEF increased significantly with the percentiles of the trait distribution (Figs. 6A and 6B). At the 90th percentile, *h*^2^** was 44% greater than at the 10th percentile (0.675 ± 0.060 vs. 0.468 ±0.046). This was largely due to FEF75% (Figs. 7A and 7B). Specifically, those whose expiratory force was high relative to the population (90th percentile) were more strongly associated with their parents and siblings than those whose force was relatively low (10th percentile, i.e., regression slope 80% and 70% greater for parent offspring and sibs, respectively). In contrast, offspring-parent and full-sib regression slopes were relatively constant at FEF25%.

## Discussion

Prior studies tacitly assume that the genetic and non-genetic factors affecting pulmonary function in parents, offspring, and other related individuals apply to all quantiles of the phenotype. This assumption appears valid for FVC and PEF, and generally applies to FEV_1_. It does not appear valid for the FEV_1_/FVC ratio, whose heritability at the 90th percentile of the population distribution (*h*^2^** ± SE: 0.191 ± 0.025) was about one-half of that at the 10th percentile (*h*^2^** ± SE: 0.350 ± 0.060). Quantile-specific effects were also significant for full-sib association. These quantile-specific effects are a departure from the underlying statistical models used in the analyses of twin pairs, and pedigrees, segregation analyses, and GWAS.

### Implications regarding gene-environment interactions

Figures 1 and 2 suggest that estimates of genetic heritability, cultural transmission, or shared environmental are likely to increase with decreasing levels of the FEV_1_/FVC ratio. Correspondingly, environmental factors that tend to distinguish normal vs. abnormal pulmonary function such as smoking and air pollution will likely manifest differences in genetics, cultural transmission, or shared environment in accordance with the Figures. In this regard, it is hypothesize that the environment (e.g., smoking) lowers the FEV_1_/FVC ratio, which in turn increases the phenotypic expression of genetic traits (quantile-dependent expressivity: environment →phenotype →gene expression) rather than the more traditional interpretation of gene-environment interaction (environment →gene expression →phenotype) (Williams, 2012). This scenario offers an alternative to the interpretation of several published reports purporting gene-environment interaction for pulmonary function.

Aschard et al. (2017) analyzed the association of a 26-SNP genetic risk with FEV_1_/FVC by smoking status in 50,047 participants of European ancestry from 19 studies. They found that the difference in FEV_1_/FVC between individuals who currently or previously smoked versus those who had never smoked was significantly greater if they had a high genetic risk score (GRS). They interpreted this association as a gene-environment interaction, with the susceptibility of the FEV_1_/FVC ratio to smoking being significantly greater among individuals who are genetically predisposed to having a low ratio. They proposed that the GRS could be used “to identify subgroups in the population whose genetic background makes them more susceptible to the deleterious effects of smoking”. Moreover, in the “public health setting, programmes targeting smoking cessation campaigns to individuals who are genetically predisposed to low pulmonary function may have a stronger impact in preventing COPD.”

Aschard et al. (2017) interpreted their cross-sectional association as an effect of the GRS on the consequence of smoking. In their data, there was a significantly greater reduction in the FEV_1_/FVC ratio per increment in the GRS in individuals who currently or previously smoked (*β*_*GRS*_ =  − 0.0462) than in those who had never smoked (*β*_*GRS*_ =  − 0.0363, *P*_interaction_ = 5.7 × 10^−4^). Figure 1B shows that the heritability of the FEV_1_/FVC ratio increases with decreasing percentiles of the FEV_1_/FVC ratio in the population. On average, those who currently or previously smoked in the Framingham Cohorts had significantly lower FEV_1_/FVC ratio than those who had never smoked. Therefore it is speculated that smoking lowers the FEV_1_/FVC ratio, which in turn increases the expressivity of the GRS vis-à-vis its effect in those who have never smoked.

The best-documented inherited effect on pulmonary function is AAT deficiency (Global initiative for Chronic Obstructive Lung Disease, 2020). AAT is an anti-inflammatory protease inhibitor (Pi) of neutrophil elastase that is encoded by the SERPINA1 gene. Neutrophil elastase breaks down elastin in the lung parenchyma. Isoelectric focusing may be used to genotype the SERPINA1 gene, including the PiMM genotype having normal AAT concentrations, the PiMZ genotype with mild AAT deficiency, and the PiZZ genotype with severe AAT deficiency (Global initiative for Chronic Obstructive Lung Disease, 2020).

Mehta et al. (2014) examined the interaction between the SERPINA1 PiMZ and PiMM genotypes and concentrations of outdoor particulate matter of ≤10 mm (PM10) on changes in lung function over time. They reported that higher PM10 particle concentrations produced significantly greater declines in the ratio of FEV_1_/FVC in PiMZ genotypes (*β* =  − 0.003) than PiMM genotypes (*β* = 0.001), representing a significant gene-environment interaction (*P* = 0.03). Alternatively, their results could also represent larger differences in the FEV_1_/FVC ratio between the PiMZ and PiMM genotypes with increasing particle concentrations (i.e., decreasing FEV_1_/FVC ratio), reflecting significant quantile-dependent expressivity.

Bronchial hyper-responsiveness predicts declining pulmonary function and is present in approximately two-thirds of patients with non-severe COPD (Tashkin et al., 1996). Sigsgaard et al. (2000) reported that bronchial hyper-responsiveness in farming students increased with the expected severity of AAT deficiency of their Pi genotypes, but not in rural controls. They attributed this difference to the faming students’ exposure to inflammatory agents such as farm and swine confinement dust versus the rural controls’ lack of exposure to these agents. The traditional gene-environment interpretation would suggest that the effects of farm dust on bronchial hyper-responsiveness was modified by the Pi-genotype, whereas quantile-dependent expressivity would suggest that farm dust reduced pulmonary function, which in turn accentuates the genetic effect of the Pi-genotypes on bronchial hyper-responsiveness.

Quantile-dependent expressivity may also contribute to the gene-smoking interactions of the SNPs rs9862443 and rs7941377 reported by Hancock et al. (2012), and the increased effect of the FAM13A-gene haplotype on FEV_1_/FVC from 0 (*β*_*effect*_ =  − 0.51), >0 to 15 (*β* =  − 0.38), >15 to 30 (*β* =  − 0.61), and >30 pack years (*β* =  − 1.40) in Koreans (Kim et al., 2015).

### MMEF and FEF

FEF75% and MMEF both exhibited significant quantile-dependent expressivity of offspring-parent and sibling similarity. These measures may assess small airway function (Wood, Tan & Stockley, 2009), although its use in diagnosing small airway disease in individual patients is discouraged (Quanjer et al., 2014). Lung elastic recoil and pleural pressure are lower and the location of the flow-limiting segment (choke point) is further upstream in airway segments towards the end of a forced expiratory maneuver then earlier. In addition, obstructions in small airways are hypothesized to have a greater effect on airflow at low than high lung volumes (Wood, Tan & Stockley, 2009).

### Methodological issues

Offspring-parent and sibling regression coefficients are simple estimates of heritability relative to its estimation from twin studies, family studies and segregation analyses. As has been reported previously in this and other cohorts (Devor & Crawford, 1984; Coultas et al., 1991; Klimentidis et al., 2013; Redline et al., 1989; Wilk et al., 2000; Tarnoki et al., 2013; Hukkinen et al., 2011; Hallberg et al., 2010; DeMeo et al., 2004; McClearn et al., 1994; Palmer et al., 2001; Astemborski, Beaty & Cohen, 1985; Beaty et al., 1987; Lewitter et al., 1984; Cotch, Beaty & Cohen, 1990; Chen et al., 1996; Chen et al., 1997; Givelber et al., 1998; Ingebrigtsen et al., 2011; Joost et al., 2002; Tian et al., 2017; Yamada et al., 2015), these analyses showed moderate to strong heritabilities for pulmonary function. The generally greater heritabilities for the sib-based vs. offspring-parent-based estimates have been reported by others (Givelber et al., 1998) and suggest that shared environment or dominance effects might not be negligible. Table 2 presents the significance levels reported by Stata, but it is important not to interpret significance levels much less than *P* < 0.001 literally, but rather as a indicator of strong statistical significance. Although results for MMEF and FEF were statistically significant, these measurements are difficult to make and not encouraged for use clinically due to their variability. Their variability is partly technique related, which is particularly apropos given the changing Framingham methodology over the course of data collection. The calculations of offspring-parent and full-sib quantile regression presented here assigned equal weights to each offspring (Karlin, Cameron & Williams, 1981), but there may be additional clustering within family sets not addressed by these analyses.

## Conclusions

These analyses suggest that the heritability of FEV_1_/FVC ratio, MMEF, and FEF75% are strongly dependent upon whether the offspring or sib is high or low relative to its distribution in the population. Quantile-dependent expressivity may account for gene-environment interactions between pulmonary function and smoking or airborne PM10. Caution is warranted when assigning risk from smoking or PM10 concentration based on specific genotypes when in fact low FEV_1_/FVC may be the cause for greater susceptibility.

##  Supplemental Information

10.7717/peerj.9145/supp-1 Supplemental Information 1 Stata commands used within the STATA statistical package for running quantile regression

## Abbreviation Key

AAT: α-1 antitrypsin
β_*FS*_: Full-sib regression slope
β_*OM*_: Offspring mid-parental regression slope
β_*OP*_: Offspring parent regression slope
COPD: Chronic obstructive pulmonary disease
FEF25%: Forced expiratory flows at 25% of FVC
FEF50%: Forced expiratory flows at 50% of FVC
FEF75%: Forced expiratory flows at 75% of FVC
FEV_1_: Forced expiratory volume in 1 second
GOLD: Global Initiative for Chronic Obstructive Lung Disease
GWAS: Genome-wide association studies
*h*^2^: Heritability in the narrow sense
MMEF: Maximum mid-expiratory flow
PEF: Maximum speed of expiration
PM10: Particulate matter ≤10 mm
SD: Standard deviation
SE: Standard error
SNP: Single nucleotide polymorphisms

## Additional Information and Declarations

### Data Availability

The following information was supplied regarding data availability: The raw data are available at the National Institutes of Health: https://biolincc.nhlbi.nih.gov/studies/framcohort/, https://biolincc.nhlbi.nih.gov/studies/gen3/, https://biolincc.nhlbi.nih.gov/studies/framoffspring/. The code is available as a Supplemental File.
